# Self-Assessment of Hygiene Practices towards Predictive and Preventive Medicine Intervention: A Case Study of University Students in Ghana

**DOI:** 10.1155/2019/3868537

**Published:** 2019-08-04

**Authors:** Stephen T. Odonkor, Jones Kitcher, Mavis Okyere, Tahiru Mahami

**Affiliations:** ^1^School of Public Services and Governance, Ghana Institute of Management and Public Administration, Accra, Ghana; ^2^Metropolitan Research and Education Bureau, Accra, Ghana; ^3^National Blood Service, Accra, Ghana; ^4^Ghana Atomic Energy Commission, Kwabenya-Accra, Ghana

## Abstract

**Introduction:**

Personal hygiene is essential to the current paradigm shift towards predictive, preventive, and personalized medicine, which enables the prediction and prevention of infectious disease outbreaks.

**Objective:**

The aim of this paper was to evaluate the personal hygiene practices among university students aiming at providing a basis for preventive and predictive medical interventions and to make future efforts improve target interventions for young people.

**Methods:**

The study was conducted using a cross-sectional study. Validated instruments that related personal hygiene practices were used to obtain quantitative data from 412 tertiary students from seven universities in Accra, Ghana. The resulting data were analyzed with IBM-SPSS, version 23.

**Results:**

There were more female respondents (54.4%) in the study than male respondents (45.6%). Respondents between the age group of 19-24 years constituted majority (59.7%) of the respondents in the study. Respondents from urban areas exhibited good hygiene practice compared to those from urban residences. There was a significant association between residence and hygiene practice (*χ*^2^=17.8, P≤0.001). We also observed that those respondents within the upper class in society had a poor hygiene practice, compared to the Lower Class and Middle Class respondents. Lack of education (63.1%) was observed as the main barrier to personal hygiene among the respondents. Future of the society depends on the health of its youth.

**Conclusion:**

A significant number of students are not actively practicing good hygiene. There is a need for deployment of preventive medicine interventions targeted at young people. It calls for improvement in methods of hygiene education for young people in tertiary institutions and the inclusion of hygiene in school curricula.

## 1. Introduction

The United Nation's Sustainable Development Goal of good health and well-being has been embraced globally as a result of its aim of reducing mortality [1]. The potential of achieving this goal requires a paradigm shift from the traditional approach to disease prevention and treatment and education.

The quest for innovative and advanced health care has provided the paradigm change from delayed interventions to predictive medicine tailored to the person, from reactive to preventive medicine, and from disease to wellness [2–4]. Thus, Predictive Preventive and Personalized Medicine (PPPM) is emerging as the focal point of efforts in health care aimed at curbing the prevalence of both communicable and noncommunicable diseases within the global community [5].

PPPM is the new integrative concept in the health care sector that enables predicting individual predisposition before onset of the disease, to provide targeted preventive measures and create personalized treatment algorithms tailored to the person [2, 5]. The expected outcomes are conducive to more effective population screening, identification of persons at risk, and reduction of adverse health effects [4].

A key component for the success of any predictive and preventive measure will thus include a behavioral analysis of current happenings within a given population. Hygiene behaviors and practices among a given population will thus provide a great deal of insight towards the predictive and preventive medicine process.

Hygiene is an old concept related to medicine, as well as to personal and professional care practices. Hygiene refers to the set of practices linked to the conservation of health and healthy living [6, 7]. It involves practices and conditions that help to maintain health and prevent the spread of diseases as well as practices that deal with the preservation of health [8].

Personal hygiene in a straight-line aids in disease prevention and health promotion [9–11]. Hygienic practices are prejudiced by social, familial, and individual factors as well as the individual's knowledge and attitudes towards hygiene [12, 13].

Regular hygienic practices may be considered good habits by a society while the neglect of hygiene can be considered disgusting, disrespectful, or even threatening [14, 15]. Maintaining personal hygiene is necessary for many reasons such as personal, social, health, psychological or simply as a way of life. Keeping a good standard of hygiene helps to prevent the development and spread of infections and disease [16]. This phenomenon therefore makes hygiene practices a great tool in predictive and preventive medicine processes. This comes at the back of the huge acknowledgement received by predictive and preventive medicine by global and regional organizations such as the Organization of United Nations, the European Union, and the National Institute of Health [4].

Prevention of infectious diseases has become one of the daunting challenges facing developing countries all over the world in varying degrees [17], with Ghana being no exception. The aim of this paper was to evaluate the personal hygiene practices among university students in Accra Ghana aiming at providing a basis for preventive and predictive medical care, with a goal to make future efforts improve target interventions for young people.

## 2. Methodology

### 2.1. Description of the Study Location

The study was conducted in the Greater Accra Region, which lies on the south-eastern part of the country. The region occupies a total land area of 3,245 sq. km, which makes it the smallest region of the 10 political regions in Ghana in terms of land size. It has a population density of 1,235.8 people per sq. km. The region is 90.5% urban with an annual urban growth rate of 3.1%. It experiences more inflow of people from other parts of the country than people moving out of the region.

### 2.2. Study Design and Sample Size

The study employed cross-sectional design to obtain quantitative data. The study was carried out in seven (7) university colleges in the Greater Accra Region of Ghana. The study population included both public and private universities. A total of 412 questionnaires were distributed across the seven (7) universities/university colleges in the Greater Accra Region of Ghana based on the proportion of the population of the university colleges.

#### 2.2.1. Sample Size Determination

The sample size was determined using Miller and Brower's mathematical formula for estimating single proportions [18]. The standard normal deviation was set at a 95 % confidence level, prevalent with the allowable margin of error of 0.08. The formula n = N/ 1+N(*α*)^2^ was used to determine a sample size for each university. The minimum sample size increased and rounded up when 10 % of the calculated, minimum sample size was added for nonresponse, inappropriately filled or missing questionnaires since the questionnaires were interviewer administered. In the formulae: n = Sample Size, N = Total Population, and *α* = Margin of Error, adopted from Miller and Brower [18].

### 2.3. Sampling Technique

The study utilized a stratified sampling technique. The total number of respondents in the seven (7) university colleges was obtained through a proportional sampling to size method. Thus, in selecting the respondents, sampling proportionate to size was used to determine the number of students to be interviewed from each university. At the university, all students who were present at the university were considered for the study.

### 2.4. Data Collection and Analysis

The study took place between September 2018 and December 2018. A standardized structured questionnaire designed to meet the objectives of the research was used for data collection. Field inspection of questionnaire data was carried out days after the interview was conducted, and any errors were immediately verified and corrected. The survey instrument comprised 34 questions, which elicited information on sociodemographic characteristics, including age, gender, marital status, social status, and accommodation settings. The final instrument comprised the six major areas: sociodemographics (12); oral hygiene (2 items); nails hygiene (3 items); bathing hygiene (2 items); attire and underwear hygiene (4 items); hair care (2 items); hand washing (8 items); and barriers to hygiene (1 items).

The options were weighted none = 1, sometimes = 2, and regularly = 3. Mean (x) and standard deviation (SD) were calculated for the purposes of description and for answering the research questions. The following criteria were used to interpret the results of the study: a mean (x) of 2.01-3.0 implied that students adopted a Good hygiene practice (GP); 1.01-2.0 implied that students adopted Moderate hygiene practice (MP), and 0.1-1.0 implied that students adopted Poor hygiene practices (PP). Because the scale used is an ordinal variable, the median scores were used to test the differences between each group on key variables, i.e., age, gender, programme of study, and accommodation settings.

Five experts in health education and measurement and evaluation determined face validity of the instrument. The average overall face validity was equal to 95%. Test-retest reliability was done by Alpha (Cronbach's) test reliability for internal consistency and it was equal to the reliability coefficient of 0.87, which is adjudged high reliability. It took approximately 25–35 minutes to complete the instrument.

#### 2.4.1. Ethical Considerations

Prior to data collection, respondents' verbal consent was sought. Respondents were informed about the purpose of the study and were made to understand that participation was voluntary and refusal to participate in the study would not affect their employment status. The study respondents were assured of confidentiality and informed that they could withdraw from the study at any time and were at liberty not to answer any question they did not want to. All respondents were advised that completing the survey implied informed consent to use the data for research purposes. In addition, all personal identifiers were removed in the summary data to ensure confidentiality.

#### 2.4.2. Data Handling and Analysis

Data were entered into a spreadsheet and later exported to SPSS version 23 and coded for analysis. The analysis included both descriptive and inferential statistics. Descriptive statistics (frequencies, means, and standard deviations) were used to describe the variables of interest. Univariate analysis was used in obtaining the frequency of sociodemographic characteristics and other discrete variables of the study population. Data were analyzed by contingency table except for t-tests as appropriate for continuous data (for example, age). The Chi Squared (X^2^) tests were used for assessing the bivariate relationships between these factors as well as for differences in proportions and for other categorical variables. The Fisher's exact test was used when the minimum expected frequencies were less than five in a* 2 x 2* table. Cramer's V exact test was used to determine the strength of relationships. Post hoc analysis in Chi Square was also carried out [19, 20]. All statistical tests were two-tailed and alpha = 0.05 or less was considered statistically significant.

## 3. Results

Table 1 shows the sociodemographic characteristics of the respondents. There were more females (54.4%) in the study than males (45.6%). Respondents between the age group of 19-24 years constituted 59.7% of the respondents in the study. Age group 25 and above were the least (19.2%). Christians constituted 93% of the respondents by way of religious background; Islam followed this with 5.8% and other religions with 1.2 %. The Akan ethnic groups had 55.1% respondents followed by Ga-Adangbe with 19.9%. Ninety-two (92%) percent of the respondents are single, 7.7% were married, while 0.7 % were divorced. Undergraduate students constituted more than half of the respondents (50.5%); the least student groups were postgraduate students (6.6%). In terms of students programme of study: business students constituted 36% followed by 27.2%, 18.2%, 14.1%, and 3.6 % for students in the Arts/Social Sciences, Law and Sciences respectively. With the social status of the respondents, it can be observed that 68% belonged to the middle class while 24% and 7% belonged to the Upper Class and Lower Class respectively. Furthermore 83.7% of the respondents lived in urban residences while 16.3% lived in rural residences.

Table 2 presents respondents' hygiene practices in relation to the various questions that were asked. From the table, it can be observed that teeth brushing was highest and best hygiene practice item, recorded 84.2%, 15%, and 3% for good practices, moderate practice, and poor hygiene practices, respectively.The poorest (25.6%) hygiene practice observed was nose picking. Furthermore only 64.4% of respondents used handkerchiefs when picking their nose. When asked whether they washed their hands on their return from school, only 48.3% had a good hygiene practice with this, and 11.2 % showed poor hygiene practice with this.

Table 3 shows the respondents options on the barriers to personal hygiene. Two hundred and sixty (260) respondents representing 63.1% listed lack of education as the main barrier to personal hygiene. This was followed by 20.0%, 12.7%, and 3.5% for laziness, lack of time, and inadequate water supply, respectively. Only 2 male respondents representing 0.5% listed religious beliefs as a barrier to personal hygiene.

Table 4 shows the correlation between hygiene practice and selected demographic variables. From the table, it can be observed that hygiene practice correlated with age, ethnicity, level of study, social status, marital status, and type of residence. However, it is worth noting that ethnicity, marital status, and residence was positively correlated whiles age, level of study, and social statuses were negatively correlated. No correlation was observed between hygiene practice and religion, gender, programme of study, and health insurance.

Table 5 shows the relationship between hygiene practice, gender, and residences of respondents. It can be observed that females had a significantly good practice and moderated practice of 83.3% and 14.5% respectively, while the minority (3.2%) of females had a poor practice, but was not significant (P=0.114). Similarly males represented 76.1% for good practice and 39% moderate practice. Males had relatively poor practice compared to females, however, that was also significant (P=0.114). There was a significant relationship between residences and hygiene practice (P ≤ 0.001). However, respondents from rural residence had significantly poor (P ≤ 0.001) hygiene practice compared to those in the urban residence. There was no significant association between the various age groups and their hygiene practices.

Table 6 shows the relationship between hygiene practice, social status, and insurance. The table reveals a significant relationship between hygiene practice and insurance and social status. Noninsured respondents had higher good hygiene practice (81.1%) than insured respondents (79.3%). However noninsured patient had a significantly poor hygiene practice (P ≤ 0.001) compared to insured respondents. Interestingly, the Upper Class respondents have the poorest hygiene practice (15.4%) compared to 4 % and 1.1% for Lower Class and Middle Class, respectively.

Table 7 shows the relationship between hygiene practice, programme of study, and level of study. There is no relationship between the respondents' programme of study and their hygiene practices. However, there is a significant relationship (P ≤ 0.001) between the qualifications the students were enrolled for and their hygiene practices.

## 4. Discussions

Self-assessment of hygiene behavior and life styles is not only an important determinant of the generation of accurate disease burden estimates among target populations but also is critical towards preventive and predictive medicine [21–23]. The aim of this paper is to evaluate the personal hygiene practices among university students in Accra, Ghana, aiming at providing a basis for preventive medical intervention, with a goal of making future efforts improve target interventions for young people. Personal hygiene among the youth is essential as it forms part of their developmental stages and contributes to the general well-being and health of the individual [12]. During the adolescence stage, self-care activities become more important as the body begins to mature and physiologic changes start to occur [24]. Personal hygiene practice is affected by many factors which are the developmental level, cultural background, socioeconomic status, personal habits, and health status [25, 26]. 

In this study, we found that a significant number of respondents engaged in good hygiene practice for all the described activities (Table 2). Basic personal hygiene refers to the principle of maintaining cleanliness and grooming of the external body. It includes practices like bathing regularly, washing hands whenever necessary, trimming finger and toe nails, wearing washed clothes daily, washing the hair, keeping hair clean from lice and dandruff, brushing the teeth, and caring for the gums [27]. This according to WHO is the basis for good personal hygiene [28].

The personal hygiene practices that appeared to be generally strong among study participants included washing hands after using the toilet (76.2%), brushing teeth at least once a day (84.2%), washing hands before eating (73.3%), and bathing daily (80.1%).

In this study, most of the respondents were within the ages of 19-24 years. Out of a total of 412 students 246 were in this age group with 87 below 18 and 79 above 25 years. The majority of the respondents have therefore just been over the adolescent stage. It is expected that this majority group have learnt and are able to apply the principle of personal hygiene at the university. However, significant proportion of the respondents engages in bad hygiene practice (Table 2). This calls for concern because in Sub-Saharan Africa communicable disease outbreaks are common with devastating effects. It is therefore important that these young people are targeted with preventive medicine interventions to help improve their personal hygiene practices thus reducing disease outbreak which might emanate from their poor personal hygiene [29].

The study also assessed (Table 5) the relationship between hygiene practice, gender, and residences of respondents. The majority of the students were Middle Class and lived in the urban area as shown in Table 1. However, the results showed significant influence of urbanization on good hygiene behavior of the students. Eighty three percent (83%) of the respondents from urban areas practiced good hygiene behaviors, while 61.8% from rural residences did the same. However, 7.2% respondents from rural settlements exhibited a poor hygiene practice as against 1.7% from urban areas. External and internal resources are known to influence personal hygiene practices [30]. For example, there is a challenge with the provision of water and other sanitation needs within rural residence; these could have an effect on hygiene practices and may account for the difference in the observations of hygiene practices between respondents in rural and urban areas. Other factors also include housing condition and the ability to purchase self-care products [30].

It is worth noting that research conductor elsewhere in Africa revealed that the poor state of hygiene and sanitation services in Niger was responsible for the prevalence of waterborne diseases, which was the cause of 14% of all childhood deaths in the country USAID [31]. Similarly, WSP [32] estimated that about 121, 800 Nigerians, including 87, 100 children under age die annually from diarrhea, of which about 90% of the deaths are directly attributed to inadequate hygiene and sanitation services. In addition, it is noted that “poor sanitation is a contributing factor-through its impact on malnutrition rates-to other leading causes of child mortality including malaria and measles” [32, 33].

Interestingly, we observed females had a significantly good practice compared to males (Table 5). It is of a general knowledge that females are more hygiene conscious than males. This could have been the reason for the observation in this study. We also observed that those respondents within the Upper Class in society had a poor hygiene practice, compared the Lower Class and Middle Class respondents.

The result from this study indicates that the most significant barrier to personal hygiene from the perspective of the respondents is lack of education (63.1%). This is followed by laziness (20.0%) and lack of time (12.17%). This calls for a strategic preventive medicine intervention to address this observation. This is because the high burden of communicable diseases such as diarrhea is usually associated with poor hygiene practices. This may be a threat on the public health agenda in Ghana. Good personal hygiene practice is necessary to reduce mortality and morbidity. Preventive interventions should include public education targeted at young people as well as the addition of personal hygiene to curriculum right from basic school education, targeted at improving personal hygiene practice among Ghanaians.

The current study (Table 4) also evaluated the correlation between hygiene practice and selected demographic variables. We observed that hygiene practices were positively correlated with ethnicity, marital status, and residence. Hygiene correlation with ethnicity as observed in this study agrees with a similar work done by Anderson et al. [34]. However, the correlation with ethnicity may be explained within the context of Ghanaian culture. A key feature of Ghanaian ethnic tribes is the emphasis on personal hygiene and community cleanliness and sanitation; thus, one should therefore expect a correlation between personal hygiene and respondents who keep strong ethnic ties. It is also worth noting that, in general, there is a challenge with the provision of water and other sanitation needs within rural residence; these have an effect on hygiene practices.

Hygiene and sanitation have a direct impact on development and economic benefits. Poor hygiene and sanitation cause economic losses associated with the direct costs of treating sanitation-related illnesses and lost income through reduced or lost productivity. Poor hygiene and sanitation accounts for the heaviest existing disease burdens worldwide [35, 36]. Diarrhoeal diseases are the most common hygiene- and sanitation-related diseases accounting for about 1.7 million deaths globally every year mostly in developing countries [37] (WHO, 2009).

However improved hygiene and sanitation comes with several economic benefits, which included direct economic benefits of avoiding illnesses (the amount of money that is saved from healthcare expenses); indirect economic benefits, which included a decrease in work days lost to illness and a longer lifespan, because these benefits enabled people to work more; and (3) nonhealth benefits such as time [37–39].

## 5. Expert Recommendation

Evidence adduced in this study is compelling and provides some important answers and more importantly has relation to predictive markers that can be suggested, preventive measures (particularly the targeted population, i.e., university students) that can be effective and advised to society, and personalized interventions.

From this study, a number of predictive markers can be used to predict the poor hygiene and the subsequent possible occurrence of disease among young university students. Lack of education about hygiene appears key in predicting poor hygiene practices, similarly inadequate water supply, and sheer laziness to comply with the tenets of hygiene as well as excuses for lack of time or the want of time.

Preventive measures are key to maintaining health and well-being of university students and by extension of the general populace. First, the strict compliance to hand hygiene, including washing hands with soap and water after visiting the toilet, before eating and before preparing food, is critical as it will decrease the potential of disease risks such as the occurrences of diarrheal illness. Thus, standard suggestions for handwashing and environmental cleanliness should be actively promoted.

Secondly, accompanying the hand-hygiene promotion must be recommendations for strategies to limit skin damage, in particular the consistent use of lotions to maintain skin integrity.

Thirdly, the use of antibacterial soaps on a routine basis should also be advocated to reduce the potential of infections and diseases.

Fourthly, handwashing can be facilitated by the use of alcohol-based gel hand sanitizers settings, where running water is not accessible.

Fifthly, standard kitchen practices for safe food preparation, including hand hygiene and environmental cleaning, should be emphasized.

Finally, routine environmental cleaning is an important practice that can be encouraged within homes and in the university settings.

Personalized interventions by individuals towards better hygiene may include activities such as observing better oral hygiene and keeping hands clean to avoid compromising the safety of others. Maintaining the body in good shape requires exercising and proper dieting. These will not only boost the immunity but also improve the lifestyle and the physical appearance of the body. Finally, having a conscious habit of living in a clean environment reduces many health risks, infections, and diseases.

## 6. Limitations

The study utilized a cross-sectional design, which may present difficulties in ascertaining the direction of causality between the variables analyzed. Therefore, caution needs to be taken in the interpretation of the findings with regard to causality. The study might be vulnerable to reporting bias, response bias, and selection bias. However, the authors do not think that this would be a big problem in the study because of the standardized questionnaire used.

## 7. Conclusions

Majority of the respondents were in the adolescent stage. A significant number of respondents engaged in good hygiene practice for all the described activities. Respondents from urban areas exhibited good hygiene practice compared to those from urban areas. It was also observed that those respondents within the Upper Class in society had poor hygiene practice, compared with the Lower Class and Middle Class respondents. Females had significantly good practice compared to males. We also observed that hygiene practices were positively correlated with ethnicity, marital status, and residence.

## Figures and Tables

**Table 1:** Sociodemographic characteristics of respondents.

Variable (N = 412)	N	(%)

*Age of respondents*		
18 and below	87	21.1
19-24	246	59.7
25 and above	79	19.2

*Gender*		
Female	224	54.4
Male	188	45.6

*Religion*		
Christianity	383	93.0
Islam	24	5.8
Traditional/Others	5	1.2
Others		

*Ethnicity*		
Akan	227	55.1
Ga-Adangbe	82	19.9
Mole-Dagbon	9	2.20
Ewe	51	12.40
Others	43	10.40

*Marital Status*		
Single	380	92.20
Married	29	7.0
Divorced/separated	3	0.7

*Level of Study*		
Diploma	177	43.0
Undergraduate	208	50.5
Postgraduate	27	6.6

*Programme of Study *		
Business	152	36.9
Law	75	18.2
And other respectively	58	14.1
Arts/social sciences	112	27.2
Other	15	3.6

*Health Insurance *		
Yes	364	79.1
No	48	20.9

*Kind Insurance (N=364)*		
NHIS	326	89.6
Private Insurance Scheme	38	10.4

*Social Status*		
Upper Class	99	24.0
Middle Class	284	68.9
Lower Class	29	7.0

*Residence*		
Rural	67	16.3
Urban	345	83.7

**Table 2:** Respondent hygiene practices.

Description	Good	Moderate	Poor
	Practice	Practice	Practice
	No. (%)	No. (%)	No. (%)
Do you brush your teeth daily?	347 (84.2%)	62 (15%)	3 (0.7%)
How often do you cut your nails?	196(47.6%)	202(49.0%)	14(3.4%)
Do you take your bath daily?	330(80.1)	72(17.5)	10(2.4%)
Do you wear washed attire daily?	317(76.9%)	82(19.9)	6(1.4%)
Do you iron your attire before wearing?	236(57.3%)	160(38.8%)	16(3.9%)
How often do you change your underwear?	254(61.7%)	135(32.8%)	21(5.1)
How often do you remove unwanted hair?	245(61.7%)	135(32.8%)	5(10.2%)
How often do you wash your hair?	273(66.3%)	98(23.8%)	41(10%)
Do pick your nose?	130(31.6%)	177(43.2%)	105(25.6)
Do you use a handkerchief when picking your nose?	249(60.4%)	109(26.5%)	54(13.1%)
Do you wash hands before eating?	302(73.3%)	100(24.3%)	10(2.4%)
Do you use soap to wash hands after using the toilet?	314(76.2%)	74(18%)	22(5.8%)
Do you wash your hands when you return from school?	199(48.3%)	167(40.5%)	46(11.2%)
Do you wash your hands after blowing and wiping nose?	199(48.3%)	163(39.6%)	50(12.2%)
Do you wash your hands after handling live animals?	242(58.7%)	135(32.8%)	34(8.5%)
Do you wash your hands before touching genitals?	194(47.1%)	117(28.4%)	101(24.5%)
Do you wash your hands after touching genitals?	288(69.9%)	90(21.8%)	34(8.2%)

**Table 3:** Barriers to personal hygiene.

Variable	Male		Female		Total	
	N	%	N	%	N	%
Lack of education	153	37.1	107	26.0	260	63.1
Inadequate water supply	12	2.8	3	0.7	15	3.5
Lack of time	48	11.7	4	1.1	52	12.7
Religious beliefs	2	0.5	0	0.0	2	0.5
Laziness	75	18.2	7	1.8	82	20.0

**Table 4:** Correlation between hygiene practice and selected variables.

Sr. No	Variables	Pearson Correlation
1	Age	-0.004
2	Gender	0.149∗∗
3	Religion	0.185∗∗
4	Ethnicity	0.042
5	Level of Study	-0.003
6	Programme of Study	0.136∗∗
7	Health insurance	0.099∗
8	Social status	-0.034
9	Marital Status	0.005
10	Residence	0.043

∗ Correlation significant at P<0.05 level (2tailed).

∗ ∗Correlation significant at P<0.01 level (2tailed).

**Table 5:** Relationship between hygiene practice, gender, and residences.

*Variable*	*Hygiene Practice*						*Significance Test* *P value*
	*Good Practice*		*Moderate Practice*		*Poor Practice*		
	N	%	N	%	N	%	
*Gender *							X^2^ = 3.28
Female	184	83.3	32	14.5	5	2.3	P=0.114
Male	143	76.1	39	20.7	6	3.3	Cramer's V=0.090

*Residence*							X^2^ =17.8
Rural	42	61.8	21	30.9	5	7.2	P ≤ 0.001
Urban	285	83.1	52	15.2	6	1.7	Cramer's V=0.208

*Age*							
≥ 18	74	85.1	13	14.9	0	0	X^2^ =7.588
19-24	194	79.2	41	16.7	10	4.1	P=0.108
≤25	59	74.9	19	24.1	1	1.3	Cramer's V=0.96

**Table 6:** Relationship between hygiene practice, social status, and insurance.

*Variable*	*Hygiene Practice*						*Significance Test* *P value*
	*Good Practice*		*Moderate Practice*		*Poor Practice*		
	N	%	N	%	N	%	
*Insurance*							X^2^ =20.581
Insured	284	79.3	69	19.3	5	1.4	P ≤ 0.001
Noninsured	43	81.1	4	7.5	6	11.3	Cramer's V=0.224

*Social Status*							
Lower Class	68	68.7	27	27.3	4	4	X^2^ =31.882
Middle Class	241	85.2	39	13.8	3	1.1	P ≤ 0.001
Upper Class	15	57.7	7	26.9	4	15.4	Cramer's V=0.198

**Table 7:** Relationship between hygiene practice, programme of study, and level of study.

*Variable *	*Hygiene Practice*						*Significance Test* *P value*
	*Good Practice*		*Moderate Practice*		*Poor Practice*		
	N	%	N	%	N	%	
*Programme of Study*							
Business	211	80.2	48	18.3	4	1.5	X^2^ =9.035
Law	42	75	11	19.6	3	5.4	P=0.804
Science	48	85.7	7	12.5	1	1.8	Cramer's V=0.045
Arts/Soc. Sciences	26	72.2	7	19.4	3	8.3	

*Qualification of Study*							
Diploma	143	81.3	30	17	3	1.7	X^2^ =1.628
Undergraduate	164	78.8	37	17.8	7	3.4	P ≤ 0.001
Postgraduate	20	74.1	6	22.2	1	3.7	Cramer's V=0.198

## Data Availability

The data used to support the findings of this study are available from the corresponding author upon request. The data used for the manuscript were safely stored and are available upon request.
